# Retrospective analysis of the diagnostic spectrum of histological nephropathies from the Magdeburg kidney biopsy cohort (MD-KBC)

**DOI:** 10.1186/s12882-025-04617-y

**Published:** 2025-12-01

**Authors:** Peter R. Mertens, Mohammed R. Kanaan, Abdul A. Abou-Watfa, Sascha T. Bender, Thorsten Wiech, Hermann-Josef Gröne, Florian G. Scurt

**Affiliations:** 1 University Clinic for Nephrology and Hypertension, Diabetology and Endocrinology, Otto-von-Guericke University Magdeburg, Leipziger Str. 44, 39120 Magdeburg, Germany; 2 Department of Urology and Urological Oncology, Hannover Medical School (MHH), Carl-Neuberg-Str.1, 30625 Hannover, Germany; 3 Institute of Pathology, Section of Nephropathology, University Medical Center Hamburg-Eppendorf, Hamburg, Germany; 4 Institute of Pharmacology, University of Marburg, Karl-von-Frisch-Straße 2, 35043 Marburg, Germany; 5 Medical Faculty, University of Heidelberg, 69120 Heidelberg, Germany

**Keywords:** Kidney biopsy, Epidemiology, Glomerulonephritis, Temporal trends, Diabetic nephropathy, Germany

## Abstract

**Background:**

The epidemiology of biopsy-proven kidney disease varies geographically, yet data from Central Germany are lacking, hindering local disease understanding and healthcare planning. We aimed to characterize the spectrum, trends, and predictors of kidney diseases in this region.

**Methods:**

We retrospectively analyzed 1,029 native kidney biopsies from a tertiary center in Saxony-Anhalt (2010–2021), assessing demographic, clinical, and histopathological data. Temporal trends were compared between two 6-year periods. Multivariate logistic regression identified predictors for major diagnoses.

**Results:**

Mean patient age was 54.3 years (65% male). Primary glomerulonephritis (GN) was the most common diagnosis (50.4%), led by IgA nephropathy (IgAN) (23.0%), diabetic nephropathy (DN) (10.0%), and vasculitis-associated GN (8.7%). Trend analysis revealed a significant decrease in primary GN (56.4% to 46.5%; *p* < 0.001) and an increase in vascular nephropathies (2.7% to 10.6%; *p* < 0.001). Male sex was an independent predictor for IgAN (OR 1.41), while younger age and female sex predicted Minimal Change Disease or Lupus Nephritis.

**Conclusion:**

This first study from Central Germany reveals a unique epidemiological profile with a high burden of IgAN, DN, and vasculitis. We observed a significant temporal shift from primary GNs towards vascular diseases, reflecting the rising prevalence of metabolic syndrome and hypertensive conditions. These findings underscore evolving challenges in nephrology and support the need for a national German kidney biopsy registry to monitor and address these trends.

**Supplementary Information:**

The online version contains supplementary material available at 10.1186/s12882-025-04617-y.

## Key learning points

What was known

This study adds

Potential impact

The epidemiology of biopsy-proven kidney disease is known to vary significantly by geography, with different dominant pathologies reported in Europe, Asia, and America.

Unlike many other European nations, Germany lacks a national kidney biopsy registry, resulting in a significant knowledge gap regarding regional disease patterns, especially in its central and eastern regions.

Public health data indicate a high regional burden of metabolic and cardiovascular risk factors in Saxony-Anhalt. The specific kidney manifestations of this had not been characterized systematically in a large biopsy cohort.

The first comprehensive, long-term (12-year) epidemiological data set of biopsy-proven kidney disease in Central Germany, establishing a high regional biopsy rate of 95.8 per million population, which is greater than in other reported German cohorts.

A detailed characterization of a unique “Central German phenotype” of kidney disease, defined by a triad of high-frequency IgA nephropathy (23.0%), diabetic nephropathy (10.0%), and ANCA-associated vasculitis (8.7%).

Clear evidence of a significant temporal shift in kidney pathology, with a decline in primary glomerulonephritis (partly confounded by the introduction of anti-PLA2R antibody testing) and a sharp, nearly four-fold increase in vascular nephropathies.

This study provides a strong, data-driven argument for the timely establishment of a national German kidney biopsy registry to monitor disease trends, understand regional disparities, and guide public health strategies.

The findings link the high rates of diabetic and vascular nephropathy directly to the regional public health crisis, informing the need for targeted prevention strategies and healthcare resource planning in Central Germany.

The analysis reinforces the indispensable role of kidney biopsies for accurate diagnosis in complex clinical scenarios, highlighting the limitations of non-invasive diagnostics for making high-stakes therapeutic decisions.

**Supplementary Information:**

The online version contains supplementary material available at 10.1186/s12882-025-04617-y.

## Introduction

Chronic kidney disease (CKD) is a major public health issue, affecting an estimated 850 million people worldwide and representing a leading cause of morbidity and mortality [1–4]. The global burden of CKD is increasing, and CKD is projected to become the fifth most common cause of years of life lost globally by 2040 [4, 5]. Within the spectrum of CKD, glomerular diseases are a significant contributor to the burden of kidney failure, often requiring renal replacement therapy [6–8]. The native kidney biopsy remains the indispensable gold standard for the definitive diagnosis, prognostication, and therapeutic management of most parenchymal kidney diseases, providing crucial histopathological information that cannot be obtained through non-invasive means [9–11].

The epidemiology of biopsy-proven kidney disease exhibits profound variability across different geographical regions. Large national and international biopsy registries in countries like Spain, Italy, the UK, Norway, and Japan have documented significant discrepancies in the prevalence of specific entities [12–16]. For example, IgA nephropathy (IgAN) is the most common primary GN in Europe and Asia, whereas focal segmental glomerulosclerosis (FSGS) and diabetic nephropathy (DN) are more dominant in the United States [17–21]. 

In Germany, the understanding of glomerular disease epidemiology is fragmented [22–25]. Unlike many other European nations, Germany lacks a national kidney biopsy registry. Consequently, available data are limited to regional reports, primarily from western and northern Germany [23, 26, 27]. Central Germany, and specifically the state of Saxony-Anhalt, represents a significant epidemiological blind spot. This knowledge gap is critical given the region’s distinct demographic profile, including a higher average age and a high prevalence of metabolic risk factors such as obesity and diabetes [28, 29]. 

To address this gap, we established the Magdeburg Biopsy database. This study leverages this resource to provide the first in-depth analysis of biopsy-proven kidney disease in Central Germany, aiming to: (1) define the patient profile; (2) determine the prevalence and spectrum of diseases; (3) analyze temporal trends over a 12-year period (2010–2021); and (4) identify predictors for the most common diagnoses.

## Materials and methods

### Study design

This retrospective analysis utilized the Magdeburg Biopsy database, which includes all native kidney biopsies performed at the University Hospital Magdeburg between January 1, 2010, and December 31, 2021. Adult patients (≥ 18 years) undergoing their first native kidney biopsy were included. Transplant and repeat biopsies were excluded. The study was approved by the local ethics committee of the Otto-von-Guericke University of Magdeburg (Leipziger Str. 44, D-39104 Magdeburg, Germany, No. 74/09), and it was conducted in accordance with the Declaration of Helsinki. All participants were fully informed of the study’s objectives and the voluntary nature of their involvement. Prior to the kidney biopsy, all participants provided written informed consent, which included permission for the use of their pseudonymized clinical data and remaining biomaterials for future scientific research, as stipulated by the statutes of our hospital.

### Data collection and disease classification

Comprehensive demographic, clinical, and laboratory data were extracted from electronic health records. All kidney biopsy specimens were processed and evaluated according to a standardized protocol by two expert nephropathologists, ensuring high diagnostic consistency. The evaluation included light microscopy, immunofluorescence/immunohistochemistry, and electron microscopy. To mitigate re-classification bias over the 12-year study period, the diagnoses of “primary nephrosclerosis” and “hypertensive nephrosclerosis” were combined into a single category termed “nephrosclerosis” for all analyses. Diagnoses were categorized into primary GN, secondary GN, vascular nephropathy, and tubulointerstitial nephropathy (TIN). For clarity, primary glomerulonephritis was defined as a disease process intrinsic to the kidney without evidence of a causative systemic disease. Secondary glomerulonephritis was defined as kidney inflammation occurring as a consequence of a systemic condition, such as lupus, systemic vasculitis, or diabetes mellitus. The classification of kidney function deterioration as “acute” or “chronic” was based on the documented clinical assessment at the time of biopsy, with a timeframe of less than 3 months defined as acute and a timeframe exceeding 3 months defined as chronic.

### Indications for kidney biopsy

To address potential selection bias and characterize the clinical triggers for biopsy in our cohort, we retrospectively analyzed the primary indications documented at the time of the procedure. Data were extracted from the electronic health records for the three main clinical indications: (1) deterioration of GFR (acute, subacute, or chronic), (2) proteinuria (including nephrotic and sub-nephrotic ranges), and (3) hematuria (microscopic or macroscopic). Each biopsy was categorized based on the presence or absence of these three indications, allowing for classification into seven mutually exclusive groups (e.g., isolated proteinuria, combined proteinuria and hematuria, etc.). The distribution of these indications across the entire cohort (*n* = 1029) was then calculated.

### Epidemiological and statistical analysis

Statistical analyses were performed using SPSS Statistics, Version 25.0. A two-sided p-value < 0.05 was considered significant. Descriptive statistics were used to summarize patient characteristics. For group comparisons, the Wilcoxon-Mann-Whitney U-test was used for continuous variables and the Chi-square test for categorical variables. The Cochran-Armitage test for trend was used to assess linear trends in diagnoses over two equal 6-year intervals (Period 1: 2010–2015; Period 2: 2016–2021). Multivariate logistic regression models were constructed to identify independent predictors for major diagnoses.

## Results

### Cohort characteristics and indications for kidney biopsy

Between January 2010 and December 2021, a total of 1029 native kidney biopsies were included for analysis. The baseline demographic, comorbidity, and laboratory data of the cohort are summarized in Table 1. The average age of the patients was 54.3 ± 15.8 years, with a male predominance (65.0%). Arterial hypertension was the most common comorbidity, present in 81.0% of patients, followed by diabetes mellitus (26.0%). The mean eGFR was 44.0 ± 30.8 ml/min/1.73 m², corresponding to KDIGO stage G3b. The leading indication for biopsy was an unclear deterioration in kidney function as determined by lowered eGFR (41.5%), followed by proteinuria and hematuria.

**Table 1:** Baseline characteristics of the cohort at the time of biopsy

Characteristics	Overall (*n* = 1029)	Male (*n* = 669)	Female (*n* = 360)	*P* Value
Age (years), mean ± SD	54.3 ± 15.8	55.1 ± 14.9	52.8 ± 17.2	0.099
Age Cohort, n (%)				
< 65 years	736 (71.5)	477 (71.3)	259 (71.9)	0.884
≥ 65 years	293 (28.5)	192 (28.7)	101 (28.1)	0.884
Body Mass Index (BMI, kg/m^2^), mean ± SD	28.4 ± 6.4	28.5 ± 5.6	28.4 ± 7.6	0.053
Comorbidities, n (%)				
Arterial Hypertension	833 (81.0)	563 (84.2)	262 (72.8)	**< 0.001**
Diabetes Mellitus	268 (26.0)	190 (28.4)	78 (21.7)	**0.018**
Type 1 Diabetes Mellitus	13 (4.8)	8 (1.2)	5 (1.4)	0493
Type 2 Diabetes Mellitus	253 (94.4)	164 (86.3)	68 (87.1)	0.039
Coronary Heart Disease	114 (11.1)	90	24	**< 0.001**
Peripheral Artery Disease	39 (3.8)	30	9	0.156
Cerebrovascular Accident	60 (5.8)	41	19	0.677
Key Laboratory Findings				
eGFR (ml/min/1.73m^2^)	44.0 ± 30.8	42.2 ± 29.2	47.4 ± 33.4	0.053
Serum Creatinine (µmol/l)	224.9 ± 190.6	243.8 ± 195.9	189.9 ± 175.5	**< 0.001**
Serum Urea (mmol/l)	12.1 ± 7.8	12.6 ± 7.6	11.1 ± 7.7	**< 0.001**
C-reactive Protein (mg/l)	24.6 ± 81.5	27.6 ± 96.8	18.8 ± 38.5	0.177
Protein/Creatinine Ratio (g/g)	3.3 ± 3.7	3.2 ± 3.7	3.5 ± 3.8	0.976
Albumin/Creatinine Ratio (g/g)	2.7 ± 3.9	2.9 ± 3.4	3.6 ± 5.3	0.814
Nephrotic-range Proteinuria (> 3.5 g/d), n (%)	338 (35.1)	216 (34.6)	122 (36.1)	0.269
Microhematuria (> 5 Ery/µl), n (%)	665 (63.7)	431 (64.4)	224 (62.2)	0.497

Abbreviations: BMI, Body Mass Index; d, day; eGFR, estimated Glomerular Filtration Rate; g, gram; kg, kilogram; SD, Standard Deviation

The primary clinical indications for performing a kidney biopsy were analyzed for all 1,029 patients (Supplementary Table S1). The most common indication was a combination of deteriorating GFR and proteinuria, which accounted for 24.3% (*n* = 250) of all biopsies. This was followed by combined proteinuria and hematuria (21.4%, *n* = 220). In total, biopsies performed for a combination of two or three indications represented 51.2% (*n* = 529) of the cohort. Isolated proteinuria was the indication in 19.4% (*n* = 200) of cases, while isolated deterioration of GFR accounted for 14.6% (*n* = 150). Isolated hematuria was the least common primary indication, leading to biopsy in only 4.9% (*n* = 50) of patients. The pattern of indications was consistent with the final diagnostic spectrum. For instance, IgA nephropathy was the most frequent diagnosis among patients biopsied for combined proteinuria and hematuria, whereas vasculitis-associated GN was significantly enriched in the group presenting with deterioration of GFR.

### Biopsy rate, incidence, and prevalence

In the hospital’s catchment area, which has a population of about 900,000, the cumulative prevalence of histologically confirmed kidney disease was 115 per 100,000 people by the end of the study period (Supplementary Table 2). The mean annual incidence was 9.6 per 100,000 people, and the mean annual biopsy rate was 95.8 per million people (pmp). A year-by-year summary shows an increasing trend in diagnostic activity over the study period.

### Distribution of histological diagnoses

An average of 12.7 glomeruli were available for histopathological evaluation per biopsy. Primary GNs were the largest group (50.4%), followed by secondary GNs (37.7%). The three most common specific diagnoses were IgA nephropathy (23.0%), nephrosclerosis (11.4%), and diabetic nephropathy (10.0%). The overall distribution of diagnoses is detailed in Table 2, with further stratification by sex and age.

**Table 2:** Distribution of histopathological diagnoses

Characteristics	Overall (*n* = 1029)	Male (*n* = 669)	Female (*n* = 360)	< 65 years	≥ 65 years
Primary Glomerulonephritis (GN), n(%)	463 (45.0)	301 (65.0)	162 (35.0)	352 (76.0)	111 (24.0)
IgA Nephropathy (IgAN)	237 (23.0)	168 (70.9)	69 (29.1)	188 (79.3)	49 (20.7)
Membranous GN (MGN)	80 (7.8)	54 (67.5)	26 (32.5)	48 (60.0)	32 (40.0)
Primary Focal Segmental Glomerulosclerosis (FSGS)	63 (6.1)	35 (54.8)	28 (45.2)	50 (79.0)	13 (21.0)
Minimal Change Disease (MCD)	42 (4.1)	17 (40.5)	25 (59.5)	35 (83.3)	7 (16.7)
Membranoproliferative GN (MPGN)	18 (1.7)	12 (66.7)	6 (33.3)	12 (66.7)	6 (33.3)
Necrotizing GN	17 (1.7)	14 (82.4)	3 (17.6)	14 (82.4)	3 (17.6)
Post-infectious GN	6 (0.6)	1 (16.7)	5 (83.3)	5 (83.3)	1 (16.7)
Secondary Glomerulonephritis (GN), n(%)	388 (37.7)	235 (60.6)	153 (39.4)	265 (68.3)	123 (31.7)
Diabetic Nephropathy	103 (10.0)	73 (70.9)	30 (29.1)	69 (67.0)	34 (33.0)
Vasculitis-associated GN	90 (8.7)	57 (63.3)	33 (36.7)	62 (68.9)	28 (31.1)
Lupus Nephritis (Lupus GN)	40 (3.9)	14 (35.0)	26 (65.0)	38 (95.1)	2 (4.9)
Secondary FSGS	40 (3.9)	24 (60.0)	16 (40.0)	30 (75.7)	10 (24.3)
Cast Nephropathy (Myeloma)	38 (3.7)	22 (57.9)	16 (42.1)	12 (32.4)	26 (67.6)
Amyloid Nephropathy	27 (2.6)	15 (55.6)	12 (44.4)	12 (44.4)	15 (55.6)
Medication-induced GN	16 (1.6)	11 (68.8)	5 (31.2)	11 (68.8)	5 (31.2)
Alport Syndrome	10 (1.0)	5 (50.0)	5 (50.0)	10 (100.0)	0 (0.0)
Sarcoidosis	9 (0.9)	6 (66.7)	3 (33.3)	7 (77.8)	2 (22.2)
Anti-GBM Disease	6 (0.6)	4 (66.7)	2 (33.3)	5 (83.3)	1 (17.7)
Other	9 (0.9)	4 (40.0)	5 (60.0)	9 (100.0)	0 (0.0)
Nephrosclerosis*, n(%)	118 (11.5)	83 (70.1)	35 (29.9)	70 (59.0)	48 (41.0)
Vascular Nephropathy, n(%)	11 (1.1)	8 (72.7)	3 (17.3)	9 (81.2)	2 (18.8)
Thrombotic Microangiopathy (TMA)	7 (0.7)	4 (57.1)	3 (42.9)	6 (85.7)	1 (14.3)
Ischemic Nephropathy	4 (0.4)	4 (100)	0 (0.0)	3 (75.0)	1 (25.0)
Tubulointerstitial Nephropathy (TIN), n(%)	44 (4.3)	30 (68.2)	14 (31.8)	30 (68.2)	14 (31.8)
Acute Interstitial Nephritis	21 (2.0)	11 (52.3)	10 (47,7)	11 (52.3)	10 (47,7)
Acute Tubular Necrosis	15 (1.5)	11 (73.3)	4 (26.7)	11 (73.3)	4 (26.7)
Chronic Interstitial Nephritis	12 (1.2)	8 (66.7)	4 (33.3)	8 (66.7)	4 (33.3)
No Classifiable Pathology, n(%)	7 (0.7)	3 (42.9)	4 (57.1)	7 (100)	0 (0.0)

* Nephrosclerosis combines primary and hypertensive nephrosclerosis as per study methodology. For the ‘Overall’ column, percentages are calculated from the total cohort (*n* = 1029). For all other columns (Male, Female, < 65 years, ≥ 65 years), percentages indicate the proportion of patients for that specific diagnosis (row) who fall into that subgroup

### Temporal trend analysis

To analyze temporal trends, the study period was divided into two periods: 2010–2015 (Period 1, *n* = 443) and 2016–2021 (Period 2, *n* = 585). A significantly higher number of biopsies was performed in the second period (*p* < 0.001). A statistically significant shift was observed: the proportion of primary glomerulonephritis (GN) decreased from 56.4% to 46.5% (*p* < 0.001). Concurrently, the proportion of vascular GNs increased from 2.7% to 10.6% (*p* < 0.001). The proportion of the combined nephrosclerosis category increased slightly but not significantly, from 9.5% in Period 1 to 12.8% in Period 2. The overview on the different time periods is provided in Table 3.

**Table 3:** Comparative analysis of patient demographics and specific diagnoses across study periods

Characteristic	Period 1 (2010–2015)	Period 2 (2016–2021)	*P* Value
Age (years), mean ± SD	53.0 ± 16.0	55.0 ± 16.0	0.10
Age Cohort (< 65/≥65 years), n (%)	330 (74.5) / 113 (25.5)	406 (69.4) / 179 (30.6)	0.08
Sex (Male/Female), n (%)	248 (64.1) / 159 (35.9)	384 (65.6) / 201 (34.4)	0.64
BMI (kg/m^2^), mean ± SD	27.9 ± 5.7	28.8 ± 6.8	0.09
Number of Biopsies, n (%)	443 (43.1)	585 (56.9)	**< 0.001**
Major Diagnostic Groups, n (%)			
Primary Glomerulonephritis	250 (56.4)	272 (46.5)	**< 0.001**
Secondary Glomerulonephritis	161 (36.3)	232 (39.7)	0.36
Vascular Glomerulonephritis	12 (2.7)	62 (10.6)	**< 0.001**
Tubulointerstitial Nephropathy	18 (4.1)	20 (3.4)	0.51
Major Specific Diagnoses, n (%)			
IgA Nephropathy	107 (24.2)	130 (22.2)	0.50
Diabetic Nephropathy	41 (9.3)	62 (10.6)	0.14
Vasculitis-associated Glomerulonephritis	37 (8.4)	52 (8.9)	0.50
Membranous Glomerulonephritis	36 (8.2)	44 (7.5)	0.24
Primary Focal Segmental Glomerulosclerosis (FSGS)	29 (6.5)	34 (5.8)	0.36
Nephrosclerosis (Combined)	42 (9.5)	75 (12.8)	0.50

Abbreviations: BMI, Body Mass Index; IgA Nephropathy, Immunoglobulin A Nephropathy; kg, kilogram; m^2^, square meter

### Autoimmune-related kidney diseases

A substantial portion of the cohort, 44.0% (*n* = 453), was diagnosed with an autoimmune-related kidney disease. The distribution of diagnoses within this group is shown in Supplementary Table 3. Patients in the autoimmune group were significantly younger and had a better mean GFR compared to patients with other diagnoses (Table 4a and 4b). Within the group of autoimmune diseases, patients with Lupus Nephritis were the youngest (mean age 45 years), while those with vasculitis-associated GN had the lowest mean GFR (26.6 ml/min/1.73 m²). Serological analysis confirmed a high prevalence of ANCA antibodies in patients with vasculitis-associated GN. PLA2R antibodies were present in 58.5% of patients with membranous GN. Significant complement consumption (C3 and C4) was observed in patients with lupus nephritis (35% of all cases) (Table 4b).

**Table 4a:** Comparative characteristics of patients with autoimmune vs. non-autoimmune kidney diseases

Characteristic	Autoimmune-mediated kidney diseases (*n* = 453)	All Other Kidney Diseases (*n* = 576)	*p*-Value
Age (years), mean ± SD	53 ± 15	55 ± 16	**0.026**
Age Cohort (< 65/≥65 years), n (%)	343 (75.7) / 110 (24.3)	393 (68.2) / 183 (31.8)	**0.008**
Sex (Male/Female), n (%)	294 (64.9) / 159 (35.1)	375 (65.1) / 201 (34.9)	0.948
BMI (kg/m^2^), mean ± SD	28.4 ± 6.6	28.4 ± 6.2	0.581
eGFR (ml/min/1.73m^2^), mean ± SD	46.5 ± 31	42 ± 30	**0.015**

Abbreviations: BMI, Body Mass Index; eGFR, estimated Glomerular Filtration Rate; g, gram; kg, kilogram; m^2^, square meter; SD, Standard Deviation

**Table 4b:** Serological markers in autoimmune and other nephropathies

	Vasculitis-associated Glomerulonephritis (*n* = 90)	Membranous Glomerulonephritis (*n* = 80)	Lupus Glomerulonephritis (*n* = 40)	IgA-Nephropathy (*n* = 237)	Others (*n* = 576)
c-ANCA positive, n (%)	38 (42.2)	1 (1.3)	0 (0)	7 (3.0)	62 (7.1)
p-ANCA positive, n (%)	52 (57.8)	0 (0)	3 (7.5)	10 (4.2)	47 (5.4)
PLA2R-AK positive, n (%)	6 (6.7)	47 (58.8)	7 (17.5)	25 (10.5)	93 (10.6)
C3 (g/l), mean	1.27	1.23	0.93	1.18	1.87
C4 (g/l), mean	0.29	0.28	0.20	0.28	1.31
Complement Consumption (Low C3 & C4), n (%)	4 (4.4)	4 (5.0)	14 (35.0)	18 (7.6)	71 (8.1)

### Subgroup and multivariate analysis

Urinalysis and renal function varied significantly by diagnosis (Table 5). Membranous GN and primary FSGS presented predominantly with a nephrotic proteinuria (proteinuria > 3.5 g/day), whereas vasculitis-associated GN presented with a nephritic sediment. Conditions such as Lupus nephritis and vasculitis often presented with an acute deterioration of GFR, while diseases such as membranous GN and diabetic nephropathy typically manifested as a chronic, progressive GFR decline.

**Table 5:** Renal function, urinalysis findings and clinical syndromes by major diagnostic group

Diagnosis	*n*	GFR (ml/min/1.73m^2^) mean (IQR)	Acute Decline (< 3 months) %	Chronic Decline (> 3 months) %	Proteinuria (%)	Proteinuria > 3.5 g/day (%)	Microhematuria (%)	Nephritic Sediment (%)
Primary Glomerulonephritis	519	50.4 (27.0-67.8)	39.5	60.5	88.5	65.7	72.3	29.8
IgA Nephropathy	237	48.3 (26.5–66.0)	36.2	63.8	85.5	56.9	77.2	23.7
Membranous Glomerulonephritis	80	58.7 (38.8–88.5)	25.0	75.0	100.0	82.5	63.7	32.5
Primary FSGS	63	51.4 (29.0–70.0)	30.4	69.6	85.7	66.7	61.9	23.8
Secondary Glomerulonephritis	392	37.2 (14.0–50.0)	61.8	38.2	89.9	63.1	58.0	33.7
Diabetic Nephropathy	103	42.3 (21.0–49.0)	40.5	59.5	89.3	72.8	52.4	33.0
Vasculitis-associated Glomerulonephritis	90	26.6 (10.0–29.0)	73.5	26.5	94.3	57.0	86.4	81.1
Lupus Glomerulonephritis	40	59.1 (42.0-101.0)	77.8	22.2	87.5	52.5	70.0	17.5
Vascular Glomerulonephritis	73	41.7 (24.0–53.0)	42.5	57.5	68.5	52.1	38.4	12.3
Tubulointerstitial Nephropathy	38	22.9 (7.9–30.6)	59.4	40.6	73.7	34.2	50.0	50.0

Abbreviations: eGFR, estimated Glomerular Filtration Rate; FSGS, Focal Segmental Glomerulosclerosis; IgA Nephropathy, Immunoglobulin A Nephropathy

Multivariate logistic regression analysis (Table 6) confirmed that men had a higher probability of being diagnosed with IgA nephropathy (OR 1.41; *p* = 0.041). Patients ≥ 65 years had a significantly higher probability of cast nephropathy; however, the wide confidence interval for this finding (OR 19.5; 95% CI, 2.58–147.25; *p* = 0.004) indicates a lack of statistical precision and should be interpreted with caution. Younger age and female sex were independent predictors for Minimal Change Disease and Lupus nephritis.

**Table 6:** Multivariate logistic regression analysis of predictors for major diagnosis

Variable	IgA Nephropathy	Diabetic Nephropathy	Vasculitis-associated GN	Primary FSGS	Membranous GN	Minimal Change GN	Lupus GN	Cast Nephropathy
	*OR (95% CI)*	*OR (95% CI)*	*OR (95% CI)*	*OR (95% CI)*	*OR (95% CI)*	*OR (95% CI)*	*OR (95% CI)*	*OR (95% CI)*
Gender								
Female	Ref.	Ref.	Ref.	Ref.	Ref.	Ref.	Ref.	Ref.
Male	**1.41 (1.05–1.97)**	1.51 (0.94–2.44)	0.86 (0.53–1.40)	0.86 (0.49–1.47)	1.06 (0.64–1.74)	**0.37 (0.19–0.73)**	**0.23 (0.11–0.48)**	0.62 (0.29–1.32)
Age								
< 45 years	Ref.	Ref.	Ref.	Ref.	Ref.	Ref.	Ref.	Ref.
45–64 years	**0.67 (0.49–0.99)** ^**a**^	**2.45 (1.33–4.52)**	**3.58 (1.71–7.51)**	0.99 (0.53–1.86)	1.49 (0.79–2.79)	**0.28 (0.13–0.60)**	**0.45 (0.22–0.95)**	6.10 (0.75–47.87)
≥ 65 years	**0.45 (0.29–0.69)** ^**a**^	1.78 (0.91–3.49)	**3.23 (1.47–7.05)**	0.81 (0.39–1.67)	1.48 (0.76–2.90)	**0.18 (0.07–0.45)**	**0.24 (0.09–0.67)**	**19.5 (2.58-147.25)**
Proteinuria								
< 300 mg/d	Ref.	Ref.	Ref.	Ref.	Ref.	Ref.	Ref.	Ref.
300–3500 mg/d	1.39 (0.88–2.22)	0.79 (0.40–1.54)	**3.07 (1.28–7.34)**	1.30 (0.55–3.02)	2.06 (0.71–5.98)	1.11 (0.12–9.87)	1.01 (0.35–2.81)	1.81 (0.52–6.34)
> 3500 mg/d	0.77 (0.46–1.28)	1.57 (0.81–3.03)	1.29 (0.50–3.33)	1.24 (0.51–3.01)	**4.96 (1.74–14.14)**	**19.37 (2.60-144.30)**	1.21 (0.41–3.51)	1.90 (0.53–6.77)

^a^ *p* < 0.05

Abbreviations: OR, Odds Ratio; CI, Confidence Interval; Ref., Reference category; IgA Nephropathy, Immunoglobulin A Nephropathy; Vasculitis-associated GN, ANCA-associated Glomerulonephritis; FSGS, Focal Segmental Glomerulosclerosis; Membranous GN, Membranous Glomerulonephritis; Minimal Change GN, Minimal Change Glomerulonephritis; Lupus GN, Lupus Nephritis; Cast Nephropathy, Myeloma Cast Nephropathy; mg/d, milligrams per day

### Effects of the COVID-19 pandemic

Our study included data from before and during the first year of the COVID-19 pandemic. An analysis showed that more biopsies were performed in the first 12 months of the pandemic (Mar 2020 – Feb 2021) than in the 12 months immediately prior (Mar 2019 – Feb 2020). This unexpected finding, which runs counter to trends reported at many other centers where procedures retarded or cancelled due to COVID-19 may reflect local factors such as changes in referral patterns or addressing a pre-pandemic backlog of cases. We found no significant change in the diagnostic spectrum of kidney diseases during this period.

## Discussion

This study provides the first comprehensive, single-center analysis of biopsy-proven kidney disease epidemiology from East Germany over a 12-year period, calculating a mean annual biopsy rate of 95.8 pmp, which is higher than in other published German cohorts [23]. Our primary finding is a significant temporal shift in the diagnostic spectrum, with a decline in primary glomerulonephritis and a sharp rise in vascular nephropathies.

### The regional context: a public health crisis in saxony-anhalt

A crucial context for our findings is the public health landscape of Saxony-Anhalt, the federal state where our center is located. The high prevalence of comorbidities in our biopsy cohort − 81% with hypertension and 26% with diabetes - is likely a reflection of a broader regional health crisis [29, 30]. 

#### Hypertension

According to the national GEDA health survey from the Robert Koch Institute, Saxony-Anhalt has a significantly higher prevalence of physician-diagnosed hypertension compared to the German national average. More recent data from the Gesundheitsatlas confirms this trend, reporting a hypertension prevalence of 32.9% in Saxony-Anhalt in 2023, placing it among the highest rates in Germany [30, 31]. 

#### Diabetes mellitus

Saxony-Anhalt consistently ranks as having one of the highest prevalences of Diabetes type 2 in Germany [29, 32]. A 2024 report from the Versorgungsatlas further confirmed that the state had the highest incidence of newly diagnosed Diabetes in the country in 2022 [32, 33]. 

#### Obesity

The landmark German Metabolic and Cardiovascular Risk Project (GEMCAS) identified Saxony-Anhalt as having the highest prevalence of both general and abdominal obesity in Germany, a finding supported by other national data from the Microcensus [34, 35]. The study found that Saxony-Anhalt had the maximum prevalence of obesity at 28.3% (95% CI 25.4–31.1), significantly higher than the national average [36]. 

This coincidence of exceptionally high prevalence of hypertension, diabetes, and obesity provides a compelling pathophysiological basis for the increase in vascular nephropathy and diabetic nephropathy observed in the later period of the study (2016–2021).

### Deconstructing trends and addressing a central paradox

Our analysis revealed a significant increase in vascular nephropathies and a stable, high prevalence of overall nephrosclerosis. This occurred during a time when national data for Germany showed improving trends in hypertension management, with decreasing mean blood pressure levels and increasing rates of treatment and control [37–41]. This creates a critical paradox: *Why is biopsy-proven hypertensive end-organ damage increasing in our regional cohort when national hypertension control appears to improve?*

The original, simplistic explanation of an “unhealthy lifestyle” is insufficient. A more nuanced interpretation must consider several non-mutually exclusive factors:

#### Regional divergence

The public health data strongly suggest that outcomes in Saxony-Anhalt are lagging behind national averages [29–32]. The benefits of improved national hypertension control may not have fully materialized in this high-risk region.

#### The overwhelming effect of metabolic disease

The renoprotective benefits of better blood pressure control may be overwhelmed by the concurrent and powerful epidemic of obesity and Diabetes type 2 [42, 43]. National data show a continued rise in obesity prevalence, and diabetes prevalence is projected to increase substantially [31, 42, 44–47]. The synergistic impact of hypertension, diabetes, and obesity likely accelerates the progression to advanced vascular nephropathy.

#### The time lag effect

There is a substantial delay, often spanning more than a decade, between the onset of risk factors like hypertension and the development of kidney damage severe enough to warrant a biopsy. The increase in vascular disease seen in the 2016–2021 period is likely an “epidemiological echo” of the cumulative damage from poorly controlled risk factors that were highly prevalent in the early 2000s, before the full impact of national improvements in hypertension management took effect [37, 38, 48]. 

#### Tertiary center selection bias

As a university referral center, our hospital likely attracts an increasingly complex and multi-morbid patient population over time. The observed trend may, in part, reflect a shift in referral patterns, where patients with more severe, treatment-resistant, or diagnostically challenging disease are preferentially sent for biopsy. This would enrich the cohort with more advanced cases of vascular disease, potentially exaggerating the trend compared to that in the general population.

Furthermore, our re-analysis of nephrosclerosis trends is critical. The initial finding of a sharp decrease in “primary” and a sharp increase in “hypertensive” nephrosclerosis was almost certainly a methodological artifact of re-classification bias [49]. Our combined analysis, showing a more stable prevalence, provides a more scientifically credible result and underscores the importance of rigorous methodology in registry studies [49, 50]. 

### The burden of autoimmune-related kidney disease

A significant finding of this study is the high prevalence of autoimmune-related nephropathies, which constituted 44.0% of all diagnoses. This proportion is consistent with data from broader European surveys (43.7%) but higher than that reported in the USA (38.9%) [12, 14–19, 21, 51, 52]. The predominance of IgA nephropathy within this group aligns with the known epidemiology in Europe. This finding underscores the crucial role of the immune system in the pathogenesis of kidney diseases in our region and highlights the importance of specialized diagnostics, including serological workups and histopathology, for accurate diagnosis and management.

### The indispensable role of biopsy and the call for a national registry

Despite rapid advances in non-invasive biomarkers, the kidney biopsy remains the gold standard for differentiating entities that share overlapping clinical features but require divergent therapies. Contemporary reviews concur that, for most glomerular diseases, serological or imaging surrogates cannot yet replace histology, particularly when treatment decisions involve high-risk immunosuppression [53, 54]. Our multivariate analyses illustrate this point: an elderly hypertensive patient with reduced eGFR may harbor hypertensive nephrosclerosis, IgAN, or ANCA-associated vasculitis - conditions whose prognoses and management range from supportive care to aggressive immunosuppression. Biopsy is equally critical for uncovering treatable non-diabetic kidney disease in patients with diabetes who present with atypical findings, as emphasized in recent position papers on membranous nephropathy and other glomerulopathies [55]. 

Finally, the study exposes a structural gap in German Nephrology: the absence of a national kidney biopsy registry. Countries that maintain comprehensive biopsy registries (e.g., Denmark, Belgium, and regional programs within Germany) have produced granular epidemiological data, identified temporal trends, and informed public-health strategies with far greater precision [23, 52, 56]. Establishing a federated German registry would harmonize data collection, capture regional disparities, monitor the impact of evolving diagnostic technologies, and ultimately improve patient outcomes across the country.

### Deconstructing the temporal trend in primary glomerulonephritis: the impact of anti-PLA2R testing

The reported temporal decrease in primary GN (56.4% in 2010–2015 vs. 46.5% in 2016–2021) requires careful interpretation, as it is likely driven by a fundamental shift in diagnostic practice rather than a true change in disease epidemiology. The primary driver of this trend is the introduction and clinical adoption of serological testing for M-type phospholipase A2 receptor (PLA2R) antibodies, a highly specific biomarker for primary membranous nephropathy (MN) [57]. Following its discovery in 2009, commercial immunoassays for anti-PLA2R became progressively integrated into routine clinical practice throughout our study period [58]. This evolution culminated in the 2021 KDIGO guidelines, which formally state that a kidney biopsy is not required to diagnose MN in patients with nephrotic syndrome and a positive anti-PLA2R antibody test [59, 60]. This change in “biopsy policy” means that in the second half of our study period, a significant and growing number of patients who would have previously undergone a biopsy for MN were diagnosed non-invasively. These patients are therefore absent from our Period 2 biopsy cohort, artificially deflating the proportion of primary GN. The observed trend is thus a powerful real-world example of diagnostic confounding. Interestingly, the proportion of biopsied MN cases in our cohort remained relatively stable (8.2% vs. 7.5%). This suggests that the patients who were biopsied for suspected MN in Period 2 may represent a more complex subgroup, such as those with atypical clinical presentations, seronegativity for anti-PLA2R, or suspicion of a secondary pathology, for whom biopsy remains essential.

### Strengths and limitations

This study’s strengths include its large, well-characterized cohort over a 12-year period and the high quality of its standardized Nephropathology. The high regional biopsy rate provides a representative view of severe kidney disease.

A critical consideration in any single-center biopsy series is a selection bias based on local biopsy practices. Our analysis of biopsy indications (Supplementary Table 1) provides transparency on this matter. The finding that the majority of biopsies were performed for severe and combined clinical presentations, such as deteriorating GFR with concomitant proteinuria, rather than for isolated or less severe abnormalities, reflects our center’s role as a tertiary referral hospital for complex nephrological cases. This indication profile aligns with the high prevalence of severe pathologies observed, such as vasculitis and rapidly progressive glomerulonephritis, and suggests that our cohort is representative of patients with significant kidney disease in the region, rather than being skewed by an unusually low threshold for biopsy.

This study has several additional limitations. First, its retrospective, single-center design limits the generalizability of our findings. Second, as with any registry study, missing clinical information is a potential source of bias and as a biopsy-based registry, our study does not include data on patients with suspected kidney disease who were managed histological diagnosis (e.g., patients with serological markers of vasculitis or membranous nephropathy who were not biopsied due to comorbidities or bleeding risk); therefore, we cannot comment on the prevalence of such cases. Third, we must acknowledge that evolving diagnostic criteria and inter-observer variability over a 12-year period are significant potential sources of bias that cannot be fully excluded without a systematic, standardized re-evaluation of all historical specimens. Fourth, the limitation of evolving “biopsy policy” must be emphasized. The introduction of non-invasive diagnostic markers, such as anti-PLA2R antibodies for membranous nephropathy, undoubtedly altered biopsy referral patterns over the study period, acting as a significant unmeasured confounder in the temporal analysis of primary glomerulonephritis. The absence of patient-level socioeconomic data prevents a deeper analysis of how social determinants of health, such as income, education, and access to care, contribute to the stark regional health disparities and the subsequent burden of kidney disease observed in our cohort. Moreover, the predominance of Caucasian ethnicity within the cohort renders it less applicable to more heterogeneous populations.

## Conclusion

In summary, our analysis of the Magdeburg kidney biopsy cohort reveals a significant shift in the epidemiology of biopsy-proven kidney disease in East Germany, with a decline in primary GNs and a rise in vascular nephropathies. A substantial burden of disease is driven by autoimmune-related nephropathies. These trends are deeply embedded in a regional context of high hypertension, diabetes, and obesity prevalence rates. Our findings underscore the critical need for regional kidney biopsy registries to monitor disease trends, inform public health policy, and highlight the consequences of the ongoing metabolic disease epidemic on renal outcomes.

## Supplementary Information

Below is the link to the electronic supplementary material.

Supplementary Material 1

## Data Availability

he datasets generated and/or analysed during the current study are not publicly available due to patient privacy regulations but are available from the corresponding author on reasonable request.

## Abbreviations

BMI: Body Mass Index
CKD: Chronic Kidney Disease
eGFR: Estimated Glomerular Filtration Rate
FSGS: Focal Segmental Glomerulosclerosis
GFR: Glomerular Filtration Rate
GN: Glomerulonephritis
IgAN: IgA Nephropathy
MD-KBC: Magdeburg kidney biopsy cohort
MGN: Membranous Glomerulonephritis
RKI: Robert Koch Institute
TIN: Tubulointerstitial Nephropathy
