# Supplementary material for: Retrospective analysis of the diagnostic spectrum of histological nephropathies from the Magdeburg kidney biopsy cohort (MD-KBC)
Source: BMC Nephrol. 2025 Dec 1;26:681. doi: 10.1186/s12882-025-04617-y (PMC12670836; doi:10.1186/s12882-025-04617-y)
Supplement: Supplementary file 1 — Supplementary Material 1 [file 12882_2025_4617_MOESM1_ESM.docx]

# Supplementary Table 1. Primary Indications for Native Kidney Biopsy in the MD-KBC Cohort (n=1029).

| Indication for Biopsy | Number of Patients (n) | Percentage of Cohort (%) |
| --- | --- | --- |
| Isolated Indications | 400 | 38.9 |
| Isolated Deterioration of GFR | 150 | 14.6 |
| Isolated Proteinuria | 200 | 19.4 |
| Isolated Hematuria | 50 | 4.9 |
| Combined Indications | 629 | 61.1 |
| Deterioration of GFR + Proteinuria | 250 | 24.3 |
| Deterioration of GFR + Hematuria | 80 | 7.8 |
| Proteinuria + Hematuria | 220 | 21.4 |
| Deterioration of GFR + Proteinuria + Hematuria | 79 | 7.7 |

# Supplementary Table 2. Annual Biopsy Rate, Incidence, and Prevalence of Biopsy-Proven Kidney Disease (2010-2021).

| Period | Year | Population of Catchment Area | Number of Biopsies | Biopsy Rate (pmp) | Annual Incidence  (per 100,000) | Cumulative Prevalence (per 100,000) |
| --- | --- | --- | --- | --- | --- | --- |
| Overall Mean (2010-2021) |  |  | 85.8 | 95.8 | 9.6 |  |
| Period 1 Summary | Mean | 906,614 | 74.0 | 81.8 | 8.2 | 24.7 |
| Period 1 detailed | 2010 | 927,646 | 57 | 61.4 | 6.1 | 6.1 |
|  | 2011 | 907,027 | 52 | 57.3 | 5.7 | 11.8 |
|  | 2012 | 902,000 | 65 | 72.1 | 7.2 | 19.0 |
|  | 2013 | 902,000 | 82 | 90.9 | 9.1 | 28.1 |
|  | 2014 | 902,000 | 58 | 64.3 | 6.4 | 34.5 |
|  | 2015 | 899,010 | 130 | 144.6 | 14.5 | 49.0 |
| Period 2 Summary | Mean | 885,878 | 97.5 | 109.9 | 11.0 | 87.8 |
| Period 2 detailed | 2016 | 896,644 | 134 | 149.4 | 14.9 | 63.9 |
|  | 2017 | 891,944 | 146 | 163.7 | 16.4 | 80.3 |
|  | 2018 | 895,666 | 93 | 103.8 | 10.4 | 90.7 |
|  | 2019 | 883,000 | 35 | 39.6 | 4.0 | 94.7 |
|  | 2020 | 876,374 | 64 | 73.0 | 7.3 | 102.0 |
|  | 2021 | 871,639 | 113 | 129.6 | 13.0 | 115.0 |

Abbreviations: pmp: per million population

# Supplementary Table 3. Distribution of Diagnoses within the Autoimmune-Mediated Kidney Disease Cohort (N=453).

| Diagnosis | n |
| --- | --- |
| Primary Glomerulonephritis |  |
| IgA Nephropathy | 237 |
| Membranous Glomerulonephritis | 80 |
| Necrotizing GN | 17 |
| Secondary Glomerulonephritis |  |
| Vasculitis-associated Glomerulonephritis | 90 |
| Lupus Nephritis | 40 |
| Sarcoidosis | 9 |
| Anti-GBM Disease | 6 |
